# Does behaviour affect the dispersal of flatback post-hatchlings in the Great Barrier Reef?

**DOI:** 10.1098/rsos.170164

**Published:** 2017-05-24

**Authors:** Natalie Wildermann, Kay Critchell, Mariana M. P. B. Fuentes, Colin J. Limpus, Eric Wolanski, Mark Hamann

**Affiliations:** 1 College of Science and Engineering, Townsville, Queensland 4811, Australia; 2 TropWATER, James Cook University, Townsville, Queensland 4811, Australia; 3 Department of Earth, Ocean and Atmospheric Science, Florida State University, Tallahassee, FL 32306-4320, USA; 4 Department of Environment and Heritage Protection, Threatened Species Unit, PO Box 2454, Brisbane, Queensland 4001, Australia

**Keywords:** Great Barrier Reef, marine turtles, flatback turtle, neritic dispersal, SLIM oceanographic model, directional swimming

## Abstract

The ability of individuals to actively control their movements, especially during the early life stages, can significantly influence the distribution of their population. Most marine turtle species develop oceanic foraging habitats during different life stages. However, flatback turtles (*Natator depressus*) are endemic to Australia and are the only marine turtle species with an exclusive neritic development. To explain the lack of oceanic dispersal of this species, we predicted the dispersal of post-hatchlings in the Great Barrier Reef (GBR), Australia, using oceanographic advection-dispersal models. We included directional swimming in our models and calibrated them against the observed distribution of post-hatchling and adult turtles. We simulated the dispersal of green and loggerhead turtles since they also breed in the same region. Our study suggests that the neritic distribution of flatback post-hatchlings is favoured by the inshore distribution of nesting beaches, the local water circulation and directional swimming during their early dispersal. This combination of factors is important because, under the conditions tested, if flatback post-hatchlings were entirely passively transported, they would be advected into oceanic habitats after 40 days. Our results reinforce the importance of oceanography and directional swimming in the early life stages and their influence on the distribution of a marine turtle species.

## 1. Background

Dispersal strategies during the early developmental stages of an organism can have a profound effect on individual survivorship, fitness and distribution range [1–5]. In the ocean, physical boundaries are scarce and ocean currents promote long-distance transportation; thus, active and directional swimming, both horizontal and vertical, is of special relevance for self-recruitment of species from plankton to fish and turtles [3,6–9]. In particular, the fate of aquatic species greatly relies on passive and active dispersal mechanisms. It is common for species to display multiple mechanisms of dispersal throughout their life cycle, adapting specific strategies to different biological and ecological requirements. Passive transport relies on external forces (e.g. wind, tides, currents) and can be regarded as an uncontrolled transport mechanism with low energetic costs for individuals [2,10]. Active dispersal results from the autonomous movement of an organism and depends on its capacity to respond to a variety of cues based on complex social and physical interactions with the environment [2].

Marine turtles begin their life at sea, after emerging from nests on land, with one to several days of hyperactive swimming, in which hatchlings constantly swim away from the coastline to reach offshore waters [11–13]. Following this period, somatic energy reserves are diminished and, until recently, it was believed that turtles of several species predominantly dispersed by drifting with the oceanic currents [14]. However, new studies have indicated that the dispersal of young turtles is not likely to be entirely passive (e.g. [15–17]). In particular, research in the Gulf of Mexico suggested that young green and Kemp's ridley turtles at an estimated age of 1 year are active swimmers in their natural environment [18]. Indeed, directional swimming during the early dispersal stages could benefit turtles when orientating themselves in the open ocean [17,19], for example, moving away from cold waters, shelf seas or strong currents [18,20], and this also improves their probability of survival and protection [12,21,22].

The swimming behaviour of turtle hatchlings and post-hatchlings varies between species [23] and could reflect different strategies to reach oceanic waters [24]. Active swimming during their early life stages could lead to very distinctive dispersal trajectories [19] and life-history patterns [14]. In south and central-eastern Queensland three species of marine turtle nest concurrently along the mainland coast and islands of the southern Great Barrier Reef (GBR). Yet, despite the three species breeding in the same geographical area, they have different dispersal patterns. Most notably, post-hatchlings of green (*Chelonia mydas*) and loggerhead (*Caretta caretta*) turtles originating from this region have an oceanic dispersal and are not commonly found in the GBR Lagoon [25,26]. In contrast, flatback (*Natator depressus*) post-hatchlings display a completely neritic (non-oceanic) developmental stage [27,28]. Their neritic stage is assumed to be mainly driven by swimming behaviour and the local water circulation [29]. Nevertheless, our knowledge of behaviour of hatchling and early post-hatchling marine turtles is generally limited to the first days, and in some cases weeks, of their life [13,22,29–31], and the dispersal strategies of post-hatchling turtles remain poorly known.

Most of our current knowledge on early swimming behaviour of marine turtles is based on laboratory experiments and oceanographic models [13,16,22,29,30,32–37]. Oceanographic advection-diffusion modelling combined with animal biology and behaviour offer a useful means of exploring the likely dispersal and migration trajectories of marine turtles [3,16,20,29,34,38–40]. Thus, we used oceanographic modelling verified by field data on the distribution of young and adult animals, to improve the understanding of dispersal of post-hatchling flatback turtles, with a special focus on how swimming behaviour can influence their dispersal patterns. We suggest that there are three main drivers that favour the neritic distribution of simulated flatback post-hatchlings under typical oceanographic conditions, namely: (i) the inshore location of nesting beaches, (ii) local water circulation and net currents, and (iii) directional swimming of post-hatchlings.

## 2. Methods

### 2.1. Study population and area

**Figure 1.**
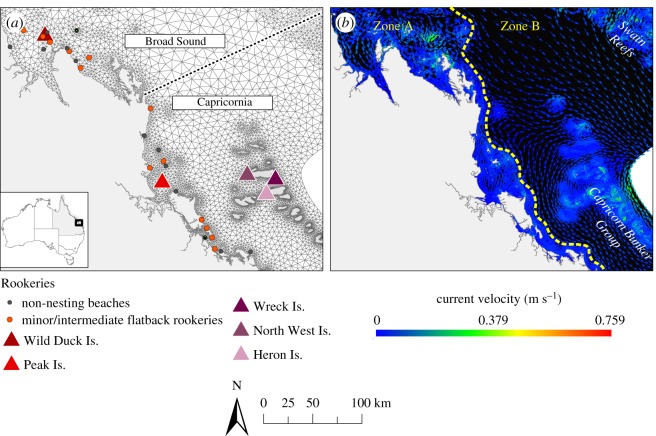
Geographical extent of the study area and model domain. (*a*) Distribution of releasing sites (rookeries) of s-turtles in each region (Broad Sound and Capricornia), background exemplifies the triangular mesh used for the SLIM model. (*b*) The net, tidal-averaged water circulation in the modelled study area. ‘Zone A’ is the wind-driven coastal boundary layer and represents areas where the currents favour a neritic dispersal, while ‘Zone B’ is the Coral Sea Lagoonal current, which flows seawards to the Pacific Ocean.

The eastern Australia flatback population nests on continental islands and mainland beaches in southern and central Queensland [27]. There are records of at least 104 rookeries (nesting beaches), the majority of them distributed within two local regions with high nesting abundance: Broad Sound and Capricornia (figure 1*a*). Two major rookeries (more than 100 nesting females/season) have been identified for this population: Peak Island located in Broad Sound, and Wild Duck Island located in Capricornia (figure 1*a*) [41]. The remainder of the nesting is distributed among intermediate (11–100 nesting females/season) rookeries, such as Curtis Island, and minor (less than 10 nesting females/season) rookeries, such as Mon Repos. Most flatback rookeries in eastern Australia are located on the continental islands within the southern GBR, and to a limited extent on mainland beaches along the central Queensland coast. There are also major rookeries for green and loggerhead turtles within the same geographical extent of the southern GBR. However, they are distributed on the outer shelf coral cays and islands of the Capricorn Bunker Group and Swain Reefs (figure 1*a*). The nesting season for marine turtles in eastern Australia begins in mid-October until late January for flatbacks, and early May for green and loggerheads; the peak emergence of hatchlings for all species is in February [27].

### 2.2. Mean water circulation in the Great Barrier Reef

The southern GBR has two areas with distinct oceanography (figure 1*b*): Zone A is located inshore and is the wind-driven coastal boundary layer where the longshore northward current is highly macro-turbulent with numerous eddies, jets and stagnation zones; Zone B is located further offshore and has a general southward current trend, which is the Coral Sea Lagoonal current formed by the intrusion of the East Australian Current (EAC) on the GBR continental shelf. This current is permanent but it waxes and wanes with the periodic formation and disappearance of the Capricorn Eddy, a cyclonic eddy which forms on the seaward side of the Capricorn channel [42–45]. The currents and the seaward water export are larger during the austral spring and summer months, in which the turtle nesting season takes place [27].

### 2.3. Methods overview

We compared the observed distribution of flatback turtles in the GBR (field evidence) to an array of scenarios in which we simulated the dispersal of turtles using a hydrodynamic advection-dispersal model. Compared to ocean simulation models, high-resolution hydrodynamic models have proved to be a useful technique to depict physical processes relevant to organismal movements in nearshore waters [5,46]. Hereafter, simulated post-hatchling turtles will be identified with an ‘s-’ prefix (e.g. s-turtles, s-flatbacks, s-greens and s-loggerheads). In a first step, we assessed the passive dispersal of the s-turtles accounting for the effect of the geographical location of nesting beaches, and the tidal phase (spring/neap) when the s-turtles entered the sea. In a later step, we added a swimming behaviour component to our simulations, and created a sensitivity analysis based on changing swimming speeds and directions, as well as the length of the passive dispersal phase.

Our dispersal simulations were based on releasing s-turtles in January 2012. This date was chosen as the climatic and oceanographic features in January 2012 were representative of the average hatching season, and the currents and wind values we used were within 1 s.d. of the average long-term conditions for the month. The variability in current velocity/direction (electronic supplementary material, figure S1*a*) and wind speed/direction (electronic supplementary material, figure S1*b*) in the southern GBR during the months that hatchlings disperse from beaches (December–February) fluctuates within a narrow range across years, with some exceptions due to extreme weather events. Thus, our modelling approach is based on assessing the variability in the dispersal due to (i) a selection of geographical/oceanographic parameters under typical climatic conditions, and (ii) a range of hypothetical swimming behaviours of the animals. The main focus of our study was to assess how behaviour influences dispersal patterns, and we acknowledge that different oceanic conditions such as monthly/yearly variability, especially those related to atypical weather events, might provide different dispersal predictions and it would be a worthy hypothesis to test in further studies.

#### 2.3.1. Direct evidence of distribution of turtles

**Figure 2.**
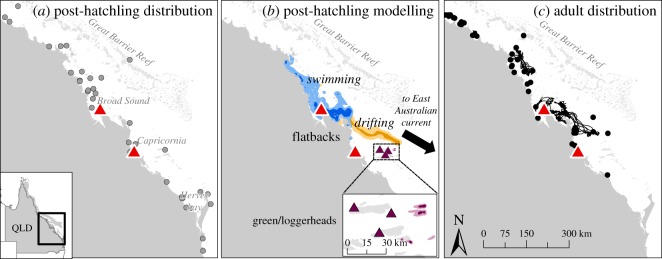
Comparison of (*a*) observed distribution of post-hatchling flatback turtles; (*b*) simulated dispersal scenarios of s-flatbacks (blue polygons: directional swimming scenario DS-1-SR, orange polygons: passive drift scenario PD-0) and s-green/loggerheads (purple polygons: passive drift scenario PD-GL-species). Polygons represent the probability of distribution of s-turtles, with dark and light colours representing 50% and 95% probability, respectively. S-flatbacks were released from Wild Duck and Peak islands (red triangles from north to the south) and were modelled for 120 days until 00 h on 2 May 2012. S-green/loggerheads were released from North West, Heron and Wreck islands (purple triangles from west to east) and were modelled for 12 days until 00 h on 14 January 2012. After 120 days of simulation, the s-flatbacks under active swimming remained inside the GBR, displaying a neritic distribution, similar to that of observed flatbacks (panels *a* and *c*). In contrast, most drifting s-flatbacks were advected seawards into the Pacific Ocean by the East Australian Current (EAC).

Data on the observed distribution of flatback turtles were used to quantitatively delineate the known distribution range of the population. Field evidence was gathered from the long-term recorded distribution of flatback post-hatchlings (*N* = 120) along the eastern coast of Australia and foraging adults (*N* = 121) from the eastern Australia stock (figure 2*a*,*c*). The database includes a comprehensive set of records based on strandings, predation, fisheries, in-water captures and satellite telemetry, between 1969 and 2016 (EHP Queensland Turtle Research database). In addition, prior to the introduction of turtle excluder devices (TEDs) there was evidence of juvenile and adult flatback by-catch in the East Coast Otter Trawl Fishery of Queensland and it occurred across most of the Queensland coast, with higher CPUE within the GBR [47]. There is no evidence of flatback turtles of any life stage outside the continental shelf of Australia. In addition, all life stages co-occur in similar regions within the inshore waters [27]. The distribution of flatback post-hatchlings has been mostly recorded along the northern coast and inshore waters of eastern Australia, with some records scattered across the southern coast. Conversely, stranded and by-caught green and loggerhead post-hatchlings originating from eastern Australia have been recorded across the Pacific [25].

#### 2.3.2. Oceanographic advection-dispersal model with animal behaviour

The model domain covered the southern extent of the GBR Lagoon (figure 1). We used the unstructured-mesh SLIM model (2D Second-generation Louvain-la-Neuve Ice-ocean Model; www.climate.be/slim) to simulate the hydrodynamics of the region and the dispersal of s-turtles [48]. The mesh size varied between 150 m near reefs and islands to 10 km in offshore waters (figure 1*a*). The SLIM model has been calibrated for the GBR by Andutta *et al.* [49–51] and Thomas *et al*. [52], and used for ecological applications including the study of the accumulation and movement of marine debris [53,54], the nearshore dispersal of flatback hatchlings [29], and the fate of fine sediment in two dimensions and three dimensions [55,56]. Forcing parameters for the model included wind stress (wind speed and direction), water circulation in the Coral Sea, and the tides and tidal currents at the shelf edge following the model calibrations for the GBR [49–52]. Daily wind data between January and April, from the period 1999–2014, were provided by the Australian Bureau of Meteorology (BOM) and averaged to calculate the monthly mean wind stress during the hatching season.

The dispersal simulation allows for directional swimming of the s-turtles, following the method of Wolanski & Kingsford [5]. Scenarios were designed to test the hypothesis of passive drift (the null scenario) and active swimming dispersal (table 1). In all scenarios, the initial period of swimming frenzy was determined from published data, namely during the first hour the s-turtles swam eastwards at 0.3 m s^−1^, and then swam for 72 h at 0.08 m s^−1^ [29,30,57–59]. After the simulated swimming frenzy, either their swimming behaviour was stopped and they dispersed passively, or they swam directionally; the parameters behind each scenario are listed in table 1. The simulations lasted for 120 prototype days.

**Table 1.** Dispersal scenarios and parameters. All scenarios included a period of swimming frenzy during the first 3 days. For the sensitivity analysis, only one parameter (italics) was changed from the standard run (DS-1-SR). PD: passive drift, DS: directional swimming, GL: geographical location, TP: tidal phase, SR: standard run, Nd: flatback (*Natator depressus*), Cm: green (*Chelonia mydas*), Cc: loggerhead (*Caretta caretta*), BS: Broad Sound, C: Capricornia, CBG: Capricorn Bunker Group.

				swimming parameters			
scenario type	scenario name	species	release locations	swimming direction	swimming speed (m s^−1^)	proportion of time swimming (%)	start of swimming behaviour (day)
passive drift	PD-GL-species	Nd, Cm, Cc	BS, C, CBG	—	—	—	—
passive drift	PD-GL-regions	Nd	BS, C	—	—	—	—
passive drift	PD-GL-minor/major	Nd	minor/ intermediate and major rookeries within BS and C	—	—	—	—
passive drift	PD-GL-non/major	Nd	non-nesting beaches and major rookeries within BS and C	—	—	—	—
passive drift	PD-TP-neap/spring	Nd	major rookeries	—	—	—	—
sensitivity analysis							
passive drift	PD-0	Nd	major rookeries	—	—	—	—
directional swimming	DS-1-SR	Nd	major rookeries	northwest	0.02	75	4
directional swimming	DS-2	Nd	major rookeries	*north*	0.02	75	4
directional swimming	DS-3	Nd	major rookeries	*south*	0.02	75	4
directional swimming	DS-4	Nd	major rookeries	*east*	0.02	75	4
directional swimming	DS-5	Nd	major rookeries	northwest	*0*.*01*	75	4
directional swimming	DS-6	Nd	major rookeries	northwest	*0*.*04*	75	4
directional swimming	DS-7	Nd	major rookeries	northwest	0.02	*25*	4
directional swimming	DS-8	Nd	major rookeries	northwest	0.02	*50*	4
directional swimming	DS-9	Nd	major rookeries	northwest	0.02	*100*	4
directional swimming	DS-10	Nd	major rookeries	northwest	0.02	75	*30*
directional swimming	DS-11	Nd	major rookeries	northwest	0.02	75	*60*
directional swimming	DS-12	Nd	major rookeries	northwest	0.02	75	*90*

The passive drift was the null scenario (scenario PD-0), in which s-turtles were left to drift with currents with no additional behaviour. To comprehensively test this hypothesis, we designed five scenarios to account for the influence of the geographical location (GL) of rookeries and the tidal phase (TP) on the dispersal of turtles (table 1). To test whether the location of rookeries of the different species results in distinctive dispersal trajectories, we compared the passive dispersal of s-turtles released from a subset of rookeries where only flatbacks (*n* = 16) and only green/loggerheads (*n* = 3) nest (figure 1*a*; electronic supplementary material, table S1). We then analysed the s-turtle dispersal within the nesting range of the EA flatback population. We tested the differences in the passive dispersal of s-flatbacks (i) between regions (*n* = 8 rookeries in each region: Broad Sound and Capricornia), and (ii) within each region, comparing the major rookeries (*n* = 1 in each region; Wild Duck Island for Broad Sound and Peak Island for Capricornia) to a subset of minor/intermediate rookeries (*n* = 7 in each region) and non-nesting beaches (proximal beaches with no records of flatback nesting, *n* = 7 in each region) (figure 1*a*; electronic supplementary material, table S1). All subsets were randomly selected with the *r. sample* function in GME 0.7.3.0 [60]. In each case 5000 s-turtles were released near the selected rookeries, on 3 January 2012 at 00.00. We used the major rookeries as a proxy for each region, namely Wild Duck Island for Broad Sound and Peak Island for Capricornia. To quantify the importance of the spring–neap tidal cycle at the time when turtles entered the sea at Wild Duck and Peak Islands (figure 1*a*, electronic supplementary material, table S1), we simulated the dispersal of 5000 s-turtles from each rookery released on two different dates: (a) at neap tide (3 January 2012 at 15.00) and (b) at spring tide (7 January 2012 at 12.00).

The effect of behaviour on the dispersal of s-flatbacks was tested through a sensitivity analysis by analogy with modelling fish swimming dispersal [5], where we tested different directions and speeds based on the currently known behaviour of flatback post-hatchlings [22,29,61] and expert opinion. A standard run (scenario DS-1-SR) was arbitrarily designed based on our best guess of what is known about the biology and swimming behaviour of flatback post-hatchlings, namely that the s-flatbacks swam north-westward at 0.02 m s^−1^ for 75% of the time (the remaining 25% of the time s-turtles drift passively). To test the sensitivity of this solution to other parameters, all other scenarios were arranged by changing one swimming parameter from the standard run (table 1). The parameters that defined the swimming behaviour of s-turtles were: (i) direction: northwest, north, south, east; (ii) speed: 0.01 m s^−1^, 0.02 m s^−1^, 0.04 m s^−1^; (iii) proportion of time s-turtles spent swimming per day: 25%, 50%, 75%, 100%; and (iv) day the post-frenzy swimming behaviour started: day 4 (immediately after the end of swimming frenzy), day 30, 60 or 90 (table 1). For all scenarios that tested the influence of the swimming behaviour we released 15 000 s-turtles near the major rookeries (Wild Duck and Peak islands), on 3 January 2012 at 00.00.

### 2.4. Outputs and data analysis

We calculated the area where 95% of the s-turtles were located (this is called the 95% distribution area), as well as core dispersal zones (area where 20% of the s-turtles were located) using the Kernel Density Estimator tool in ArcMap 10.2.2 and isopleth function in GME 0.7.3.0 [60]. We assessed the degree of overlap between scenarios with the Bhattacharyya's Affinity Index (BAI), computed with the adehabitatHR package in R [62,63]. The BAI spans from 0 to 1 (1 being 100% of overlap between two areas).

To quantify the influence of behaviour in the dispersal, we calculated the dispersal success (percentage of s-flatbacks dispersed into inshore habitats of the GBR) for each individual scenario of the sensitivity analysis (26 scenarios in total; one passive drift and 12 directional swimming scenarios for each releasing location) (table 1). Then, for each scenario we averaged the dispersal success over the two releasing locations to obtain the overall dispersal success, which we ranked based on a quantile classification as very low (≤25%), low (25.01–50%), medium (50.01–75%) and high (>75%). The inshore region was delimited by the wind-driven coastal boundary layer (see ‘Zone A’ in figure 1*b*). S-turtles located in the Coral Sea Lagoonal current (see ‘Zone B’ in figure 1*b*) were considered to get swiftly advected outside the GBR, and thus counted as distributed into offshore waters.

## 3. Results

### 3.1. Turtle dispersal under the passive drift hypothesis

The passive dispersal of s-turtles differed greatly between rookeries of the different species. Assuming passive transport for the s-green and s-loggerheads under typical oceanographic conditions, the simulations resulted in 92.5% of the s-turtles leaving the model domain and entering the EAC within 14 days. In contrast, under typical oceanographic conditions s-flatback turtles remained in lagoonal waters for 40 days before starting to leave the domain. Under most passive drift scenarios for s-flatbacks, the core dispersal zones after 120 days of simulation were located within the Coral Sea Lagoonal current flowing towards oceanic waters outside of the GBR (see ‘Zone B’ in figure 1*b*).

**Figure 3.**
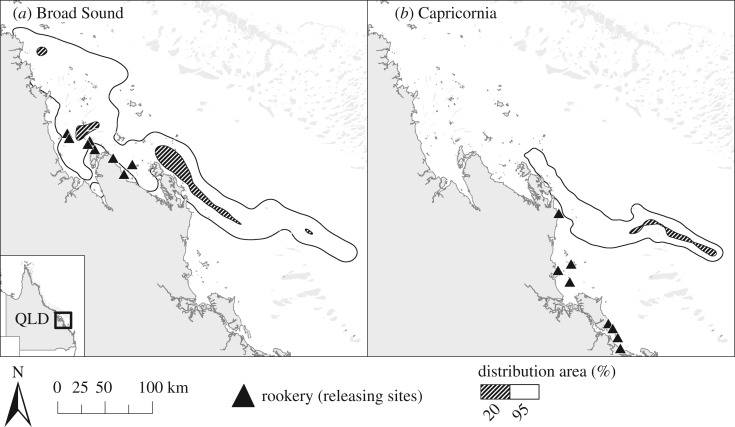
S-flatback distribution probabilities after 120 days (2 May 2012/00.00) of passive dispersal (scenarios PD-GL-regions) from (*a*) Broad Sound and (*b*) Capricornia.

**Figure 4.**
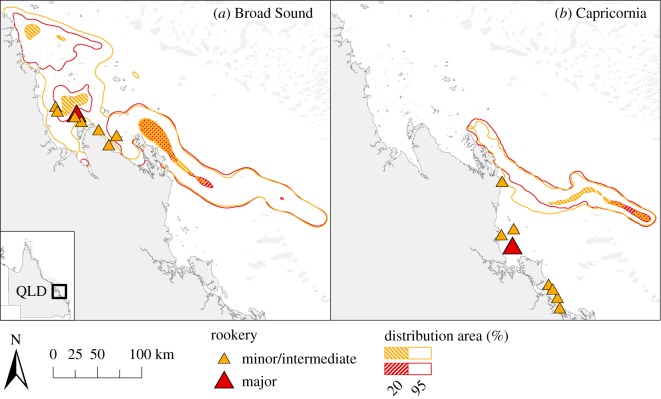
S-flatback distribution probabilities after 120 days (2 May 2012/00.00) of passive dispersal (scenarios PD-GL-minor/major) from major and minor/intermediate rookeries in (*a*) Broad Sound and (*b*) Capricornia.

In addition, s-flatback post-hatchling dispersal differed significantly between the two regions (BAI = 0.590); s-flatbacks released from Capricornia under typical conditions reach Zone B sooner and are more likely to get advected into oceanic currents (figure 3). Despite the differences between the regions, the distribution area of s-flatbacks released from non-rookeries, minor rookeries and major rookeries within each region was similar (Broad Sound BAI_minor/major_ = 0.906, BAI_non/major_ = 0.993; Capricornia BAI_minor/major_ = 0.942, BAI_non/major_ = 0.933) (figure 4).

**Figure 5.**
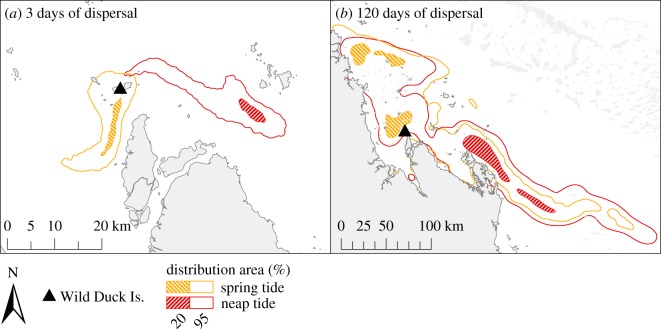
S-flatback distribution probabilities from Wild Duck Island after (*a*) 3 days and (*b*) 120 days of passive drift (scenarios PD-TP-neap/spring) after entering the sea during neap on 3 January 2012 at 15.00 and spring tide on 7 January 2012 at 12.00.

Under the conditions that were modelled, the phase of the tidal sea surface elevation (neap/spring) at the time of release had an effect on the short-term (less than 7 days of dispersal) distribution of passive s-flatbacks. Spring tides favoured a westerly dispersal of s-flatbacks by day 3 (equalling the approximate end of swimming frenzy), while neap tides dispersed s-flatbacks further away from the coast and in an easterly direction (figure 5*a*; electronic supplementary material, figure S2*a*). Furthermore, the influence of neap/spring tides on the long-term distribution of s-flatbacks varied between the releasing regions. After 120 days, for Peak Island the distribution of s-flatbacks was not affected by the phase of the tides when they enter the ocean (BAI = 0.973) (electronic supplementary material, figure S2*b*). In contrast, core dispersal zones of s-flatbacks from Wild Duck Island greatly differed between the two tidal phases (BAI = 0.000), with spring tides favouring the dispersal of s-flatbacks inside the GBR, while neap tides promoted the advection of s-turtles into the EAC (figure 5*b*).

### 3.2. The importance of directional swimming

The standard run (DS-1-SR) scenario displayed a reasonable fit with the sparse data on post-hatchling distribution (figure 2*b*). Under the passive drift scenario, 67.5% of Wild Duck Island and 94.2% of Peak Island s-flatbacks were transported seaward towards the Coral Sea after 120 days. For the standard run scenario, this loss rate reduced to 7% and 21.7% for Wild Duck Island and Peak Island, respectively. This represents between a 6- and 17-fold increase of s-flatbacks being retained inside the GBR with the addition of swimming behaviour to the simulation.

**Figure 6.**
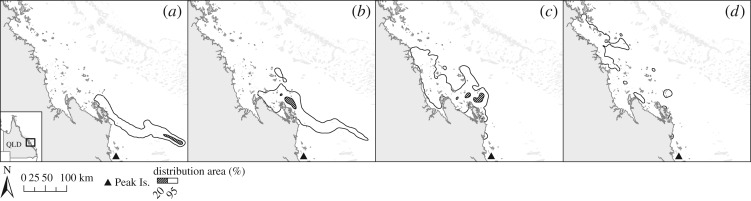
S-flatback distribution probabilities from Peak Island after 120 days at sea on 2 May 2012/00.00 under different scenarios: (*a*) passive drift (PD-0); and swimming scenarios (*b*) DS-5 (swimming speed 0.01 m s^−1^), (*c*) DS-1-SR (swimming speed 0.02 m s^−1^) and (*d*) DS-6 (swimming speed 0.04 m s^−1^).

**Figure 7.**
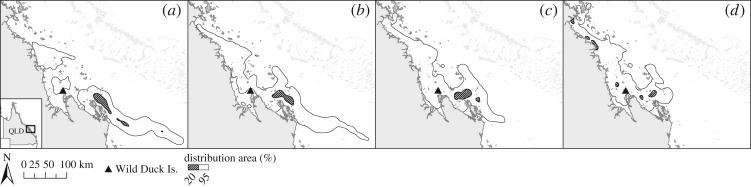
S-flatback distribution probabilities from Wild Duck Island after 120 days at sea on 2 May 2012/00.00 under different scenarios: (*a*) passive drift (PD-0); and swimming scenarios (*b*) DS-7 (swimming 25% of the time), (*c*) DS-8 (swimming 50% of the time) and (*d*) DS-1-SR (swimming 75% of the time).

The dispersal success of each swimming scenario is presented in table 2. Some of the best performing scenarios (high proportion of s-flatbacks in inshore habitats) were DS-6 and DS-3, in which turtles would swim at faster swimming speed (0.04 m s^−1^) and southwards, respectively. The horizontal swimming favouring s-turtles to remain in the coastal waters (high dispersal success) included (i) swimming at medium–fast speeds (0.02–0.04 m s^−1^, scenarios DS-1-SR and DS-6) (figure 6; electronic supplementary material, figure S3*a*), (ii) maintaining a swimming direction towards the south or northwest (scenarios DS-3 and DS-1-SR) (electronic supplementary material, figure S3*b*), (iii) developing a short initial drifting phase (start swimming between 4 and 30 days after entering the sea, scenarios DS-1-SR and DS-10) (electronic supplementary material, figure S3*c*) or (iv) spending more than 75% of the day swimming (scenarios DS-1-SR, DS-9) (figure 7; electronic supplementary material, figure S3*d*).

**Table 2.** Dispersal success (percentage of s-flatbacks in inshore waters) of each scenario of the sensitivity analysis. The dispersal success for Wild Duck Island, Peak Island, the average over the two locations and the overall dispersal success are shown for each scenario. Overall dispersal success is based on the average, and ranked as very low (≤25%), low (25.01–50%), medium (50.01–75%) and high (> 75%). S-flatbacks were modelled for 120 days until 00.00 on 2 May 2012. DS: directional swimming, PD: passive drift, SR: standard run.

	dispersal success (% of s-flatbacks in inshore waters)			
scenario name	Wild Duck Is.	Peak Is.	average	overall dispersal success
DS-4	5.61	1.8	3.7	very low
DS-2	24.03	0.0	12.0	very low
PD-0	29.14	3.4	16.3	very low
DS-12	66.61	8.6	37.6	low
DS-7	53.28	26.7	40.0	low
DS-11	81.32	25.9	53.6	medium
DS-5	66.31	44.8	55.5	medium
DS-8	76.25	55.8	66.0	medium
DS-10	88.65	70.6	79.6	high
DS-1-SR	90.99	75.4	83.2	high
DS-9	97.47	85.8	91.6	high
DS-3	87.38	98.9	93.2	high
DS-6	99.01	96.4	97.7	high

In contrast, the lowest dispersal success (low proportion of s-flatbacks in inshore habitats) was observed when s-flatbacks were entirely passive (scenario PD-0) or maintaining headings towards the east (leading s-turtles directly into the EAC, scenario DS-4) or north (leading s-turtles into the Coral Sea Lagoonal current, scenario DS-6). Low dispersal success was also related to low swimming efforts by s-flatbacks; namely, scenarios in which s-flatbacks began to swim after more than 90 days (DS-12) or spent only 25% of the day swimming (DS-7).

## 4. Discussion

This study focused on simulating, with a high-resolution hydrodynamic model, the effect of behaviour on the dispersal of s-flatback turtles in the GBR, by assessing different scenarios of passive drift and active swimming. We suggest potential mechanisms that could drive the neritic development of flatback turtles. In particular, our models indicate that s-flatback post-hatchling distribution during the first months of their life is likely to be defined by the combination of (i) inshore location of nesting beaches, (ii) local tides and net currents and, just as important, (iii) the turtle's active and directed swimming behaviour. Finally, we suggest that wind-driven waves might have an important effect in the orientation of s-flatbacks in the GBR, a hypothesis that warrants further testing.

### 4.1. The effect of nesting selection on post-hatchling dispersal patterns in the southern Great Barrier Reef

Major rookeries of green and loggerhead turtles in the southern GBR are nearly all located along the islands of the outer edge (figure 1*a*) and in close proximity to the EAC, which is the dominant oceanic current of eastern Australia. Our modelling confirmed that, even under passive drift scenarios and typical oceanographic conditions, s-green and s-loggerhead turtles are advected into the EAC within the first two weeks of life and thus are likely to have an oceanic developmental stage (e.g. [25]) (figure 2*b*). If swimming behaviour of green and loggerhead turtles is accounted for in the model, we could expect it to further facilitate an expeditious dispersal into oceanic waters. In contrast, flatback turtle nesting sites are predominately located along the islands and beaches of two regions (Broad Sound and Capricornia) of the inshore GBR (figure 1*a*) and we believe the inshore location of flatback rookeries favours the retention of s-flatbacks in the GBR lagoon during the first month of dispersal. After this period, our models indicate that s-flatbacks under typical oceanographic conditions and with no swimming behaviour eventually get advected into the EAC and exported to the ocean (figure 2*b*). It is feasible that some proportion of flatback post-hatchlings do reach the EAC, which could help explain the presence of scattered post-hatchlings records around the Hervey Bay region (figure 2*a*) and some small foraging aggregations which occur in southern Queensland.

It is also important to consider the oceanographic processes in this region, as well as their interaction with variable turtle behaviour. If the post-hatchlings were spending most of the time drifting near the surface, they would be swept directly into the EAC and dispersed into the Coral Sea. In contrast, if they were developing prolonged dives near reefs, the internal currents could increase their retention, as it happens with fish larvae and other small organisms [64]. However, if this was the case, we would expect to find more records of predated or stranded flatback turtles within the adjacent coral cays and islands of the Capricorn Bunker Group and Swain Reefs (figure 1*b*)—both of which are areas with high rates of reef-based dive and fishing operations.

Our results indicate that the advection rate of s-flatback post-hatchlings was similar among inshore major, minor and non-rookeries, as well as between tides. Combined, these results suggest that some of the evolutionary drivers that have favoured the selection of the larger flatback rookeries appear to be related to broad regional-scale differences (e.g. selection of inshore versus outer edge beaches). A previous study in the GBR suggested that nesting distribution might be related to the exposure of rookeries to wind and wind-generated waves that assist hatchlings' movement during the swimming frenzy, and indicated that flatback turtles seem to prefer sheltered beaches [65]. At a local scale, the nesting distribution of flatback turtles could also be influenced by other biophysical features (e.g. access to beach, presence of fringing reefs) which are not accounted for in our simulations.

Local water circulation patterns seem to have an important effect on the dispersal of post-hatchlings in coastal waters [29]. Based on our models, spring tides promote a nearshore dispersal during the first week and might prolong the retention of s-flatbacks in the GBR (figure 5*a*; electronic supplementary material, figure S2*a*). This finding is consistent with a previous study [29] which revealed that the tides and currents around nesting islands can influence the direction and spread of flatback hatchling dispersal during the first two weeks at sea. Distribution in nearshore areas might affect the probability of survival of post-hatchlings given that marine predators are likely to be more abundant in these areas [66,67], yet flatback post-hatchlings seem to have phenotype and behavioural adaptations which potentially decrease the risk of predation in coastal waters [22].

The region of origin also had an effect on the export/retention rate of s-flatbacks (figure 3). There was an increased seaward export of s-flatbacks from rookeries in the Capricornia region, likely to be related to the closer location of these rookeries to the Capricorn Eddy (situated around the Capricorn Bunker Group, figure 1*b*). While it has been suggested that the nesting distribution of other species of marine turtles is strongly associated with the proximity to favourable ocean currents [68], it is possible that, conversely, the nesting distribution of flatback turtles has been shaped by the selection of beaches with favourable local water circulation patterns [29], but distant from the main currents leading into the ocean.

### 4.2. Directional swimming in flatback post-hatchlings

Swimming behaviour appears to play a major role in the post-hatchlings' distribution during the first few months of their life. As mentioned before, our results provide evidence that the location of flatback nesting beaches favours the retention of s-flatback post-hatchlings during the first 40 days of dispersal. However, our simulations also suggest that under typical climatic conditions, after 40 days s-flatback post-hatchlings with no swimming are likely to be swiftly advected towards the Coral Sea into oceanic habitats where they have not been historically encountered. We tested a variety of hypothetical swimming scenarios, and conclude that directional swimming greatly decreased the seaward export of s-flatbacks, and further increased the likelihood of retention of s-flatbacks in inshore waters. This result was qualitatively validated with long-term field evidence given that, like our results, they also show that immature and adult flatbacks in eastern Australia occur within the Great Barrier Reef and coastal waters of southern Queensland (figure 2) [27,69]. Furthermore, the similarity among the modelled dispersal paths of s-flatbacks and the actual migration routes of existent tracked adult turtles (figure 2*b*,*c*) supports the theory that post-hatchlings might be able to imprint the location of habitats they pass through during their development, which can potentially influence the later migrations and distribution of juvenile and adult turtles [70–72].

In particular, s-flatbacks with active and directed swimming behaviour during the first months after entering the sea are more likely to disperse into neritic habitats. The range of horizontal swimming that favours s-turtle dispersal within the GBR (table 2; electronic supplementary material, figure S3) reveals that the more time and effort s-flatbacks invest in swimming, the higher is their chance of maintaining a neritic dispersal. Overall, our results suggest that, under typical oceanographic conditions, flatback post-hatchlings can have a short initial drifting phase, but afterwards they need to actively swim to increase the likelihood of a neritic distribution. According to our simulations, sustained speeds (more than 0.02 m s^−1^) and time spent swimming (more than 75% of the day) are influential parameters that increase the inshore dispersal success. In addition, our simulations show that accounting for directionality causes substantial differences in the dispersal outputs (table 2). For turtles released from the two major rookeries, a northwest heading (DS-1-SR) resulted in the best fit with the observed post-hatchling distribution (figure 2*a*,*b*). The scenario with a south heading (DS-3) also favoured a coastal retention of s-flatbacks, leading them to southern areas where reports of wild post-hatchlings occur but are scarce. The results of our study are based on hypothetical swimming parameters and provide valuable baseline criteria to experimentally test the orientation and swimming efforts of flatback turtles in the GBR.

While our study highlights the importance of considering swimming behaviour when simulating the early dispersal of marine turtles, numerous studies have shown that annual variability in oceanic conditions can greatly influence dispersal predictions [35,36,73]. As an example of this, simulations of the dispersal of young loggerheads in eastern Florida evidenced that not only the spread in dispersal patterns, but also the magnitude of the influence of navigational behaviour varied greatly among different years [36]. Future extensions of our modelling would be greatly enhanced by considering the influence of seasonal and inter-annual variability of the oceanographic conditions in the dispersal of post-hatchlings in the GBR.

### 4.3. Cues for directional swimming in marine species

There is a great array of sensory interactions between marine animals and their environment that probably play an important part in their behaviour, spanning from visual, olfactory or sound stimuli, to the detection of hydrodynamic and magnetic features [3,5,74]. For example, it has been proposed that coral reef fish larvae in the GBR use directional swimming to self-recruit to their natal reef, by orienting themselves using olfactory cues in open water, and auditory cues when within hearing range of their natal reef [5]. This is also the case for coastal post-larvae crab in New Zealand which use sound cues to find suitable environments to recruit [75]. The external cues that marine turtles may use to guide behaviour such as directional swimming during dispersal warrants further attention.

Directional swimming using Selective Tidal Stream Transport (STST) [7] is unlikely for turtle hatchlings because they would need to remain on the bottom for long periods to enable them to detect pressure changes and judge whether the water pressure (i.e. the tide) is rising or falling. There are, however, orientation cues that marine turtles are known to use, including the detection of wave direction during the swimming frenzy [76–78] and a combination of visual and magnetic signals to orient themselves in the open ocean [74,76,79–82]. A hypothesis that has not been tested so far is the capacity of post-hatchlings to adjust their movements based on the direction of wind-driven waves in coastal waters: for example, this direction is primarily northwestwardly in the GBR, which, if adopted for directional swimming, our simulations show favours self-recruitment of flatback turtles. The use of sound or olfactory cues remains unknown. Thus, our understanding of the early dispersal of flatback turtles would be greatly enhanced by experimentally testing the orientation of hatchlings as a response to wind-driven waves, smell/sound plumes or magnetic fields [33,80,83], or directly tracking neonates with satellite or acoustic tags [18].

## 5. Conclusion

We provide a potential explanation to the differences observed historically in the dispersal strategies (oceanic versus neritic) of coexisting marine turtle species in the GBR. The neritic distribution of s-flatback turtles was favoured by the inshore location of nesting beaches. In contrast, the outer location of reef cays and beaches, where s-greens and s-loggerheads nest, favoured a swift dispersal of these species into oceanic waters. The local water circulation in the different regions (Broad Sound and Capricornia) also seems to play an important role in the retention/advection of flatback post-hatchlings, especially during the first month of dispersal. S-turtles released during spring tides displayed a nearshore dispersal during the first week, which could have potential effects on their survival rates. Finally, under typical oceanographic conditions the inshore dispersal is further favoured if directional swimming of s-turtles is added to the models. In particular, our models suggest that high swimming efforts (in terms of speed and sustained swimming) and directionality could be important parameters to consider in future studies of flatback turtle dispersal. Our results highlight the value of integrating knowledge and applications from biological and physical sciences to improve knowledge of animal behaviour. High-resolution hydrodynamic models are a valuable tool to understand the influences of physical processes on the distribution of cryptic marine species and to infer potential pathways of dispersal [3,29,46,48]. Further advances in the resolution and numerical functions of hydrodynamic models, coupled with improved knowledge of species biology and navigation, are clearly needed. These kinds of improvements will enhance the understanding of the impact that small- and large-scale oceanographic events might have on the autonomous horizontal and vertical movements of species, and consequently on the behaviour and distribution of species, especially in view of the current changing conditions in the world's seas and oceans.

## Supplementary Material

Figure S1. Time series of (a) current velocity and direction between 1995 and 2012, and (b) wind speed and direction between 1995 and 2012

## Supplementary Material

Figure S2. S-flatback distribution probabilities from Peak Island after (a) 3 days and (b) 120 days of passive drift (scenarios PD-TP-neap/spring)

## Supplementary Material

Figure S3. Dispersal success (percentage of s-flatbacks in inshore waters) for each of the swimming parameters we evaluated in the sensitivity analysis: (a) swimming speed, (b) swimming direction, (c) proportion of time swimming per day, and (d) the day the post-frenzy swimming behaviour started. . For each scenario, the dispersal success for Wild Duck Island, Peak Island. Dispersal success are Very low (≤ 25%), Low (25.01 – 50%), Medium (51.01 – 75%) and High (> 75%). For all scenarios s-flatbacks were modelled for 120 days until 00h on 2nd May 2012.

## Supplementary Material

Table S1. Summary of releasing locations of s-turtles

## Data accessibility

The SLIM software used in this study is undergoing a licensing process at the time of publication, and is regrettably not available for public use at the present time. It is available upon request. Please contact Dr Jonathan Lambrechts (jonathan.lambrechts@uclouvain.be), and request a copy of the executable file: ‘slim-O32_January-2015.exe’. Readers are also encouraged to check with Dr Eric Deleersnijder (eric.deleersnijder@uclouvain.be) and the Catholic University of Louvain for updates on the licence availability.

The metadata used to run the hydrodynamic model and particle dispersal simulations are available at: Wildermann N, Wolanski E, Hamann M. 2017 Data Files: Metadata to run the SLIM model to simulate flatback post-hatchling dispersal in the Great Barrier Reef. James Cook University. http://dx.doi.org/10.4225/28/58dc40c459206 [84]. The dispersal simulation outputs used for the analysis are available at: Hamann, M.; Wildermann, N. 2017 Data Files: Flatback dispersal scenarios. James Cook University. http://dx.doi.org/10.4225/28/58abad9fd280a [85].
