# Supplementary material for: Does behaviour affect the dispersal of flatback post-hatchlings in the Great Barrier Reef?
Source: R Soc Open Sci. 2017 May 24;4(5):170164. doi: 10.1098/rsos.170164 (PMC5451825; doi:10.1098/rsos.170164)

Royal Society Open Science

The following supplement accompanies the article

Does behaviour affect the dispersal of flatback post-hatchlings in the Great Barrier Reef?

Natalie Wildermann[1, 2], Kay Critchell[1, 2], Mariana MPB Fuentes[3], Colin Limpus[4], Eric Wolanski[1, 2], and Mark Hamann[1, 2].

http://dx.doi.org/10.1098/rsos.170164

Author for correspondence:
Natalie Wildermann
E-mail: natalie.wildermann@my.jcu.edu.au

Figure S1a. Time series of current velocity and direction between 1995 and 2012. Grey areas represent the flatback hatching season (December – February). Current data provided by Jodie Schlaefer.

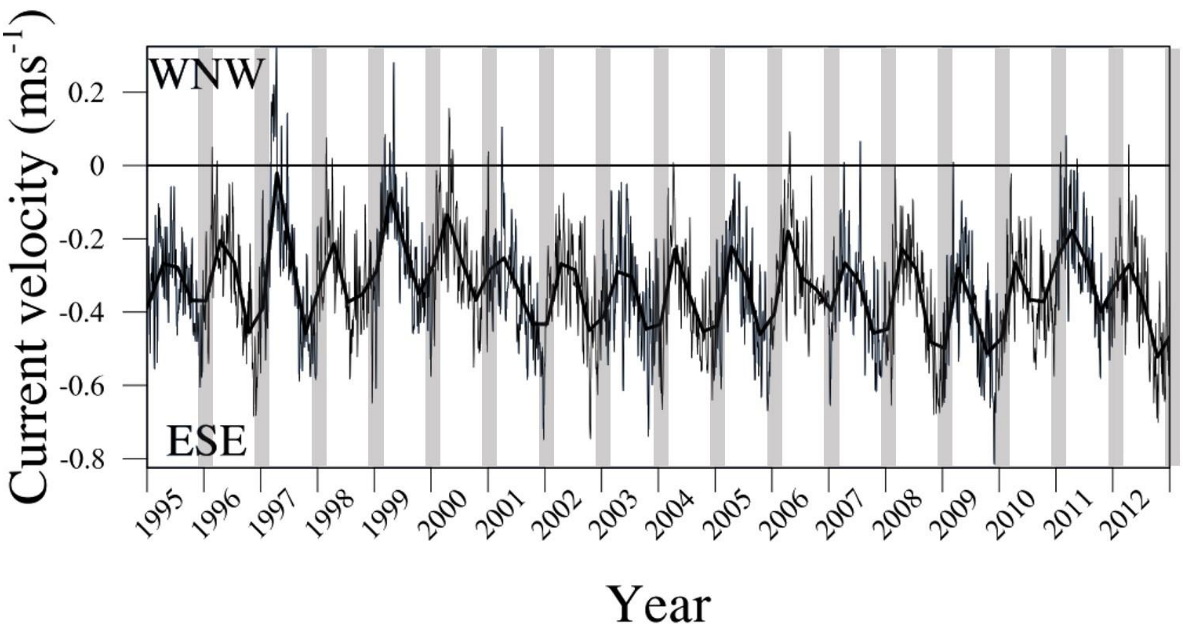

Figure S1b. Time series of wind speed and direction between 1995 and 2012. Blue areas represent the flatback hatching season (December – February). Wind data provided by the Australian Bureau of Meteorology (BoM).

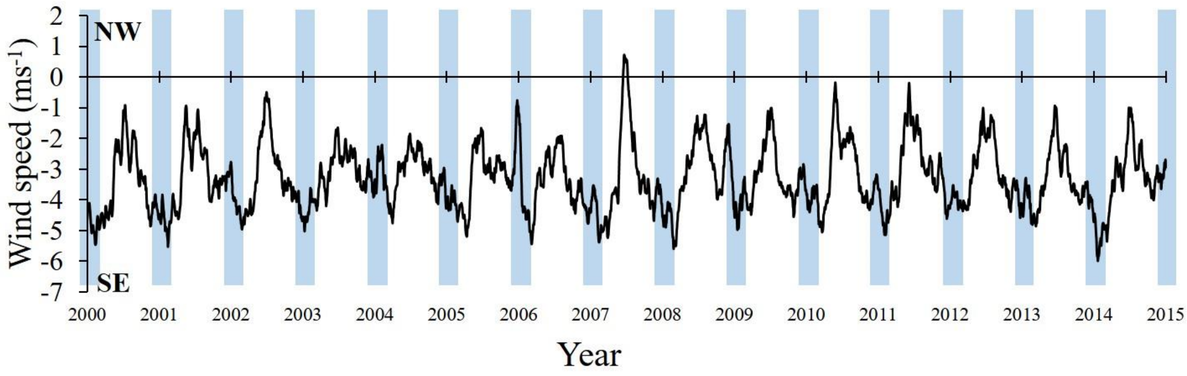

Supplement: Figure S1. Time series of (a) current velocity and direction between 1995 and 2012, and (b) wind speed and direction between 1995 and 2012 [file rsos170164supp1.pdf]
