# Supplementary material for: Does behaviour affect the dispersal of flatback post-hatchlings in the Great Barrier Reef?
Source: R Soc Open Sci. 2017 May 24;4(5):170164. doi: 10.1098/rsos.170164 (PMC5451825; doi:10.1098/rsos.170164)

Royal Society Open Science

The following supplement accompanies the article

Does behaviour affect the dispersal of flatback post-hatchlings in the Great Barrier Reef?

Natalie Wildermann[1, 2], Kay Critchell[1, 2], Mariana MPB Fuentes[3], Colin Limpus[4], Eric Wolanski[1, 2], and Mark Hamann[1, 2].

http://dx.doi.org/10.1098/rsos.170164

Author for correspondence:
Natalie Wildermann
E-mail: natalie.wildermann@my.jcu.edu.au

Figure S2. S-flatback distribution probabilities from Peak Island after (a) 3 days and (b) 120 days of passive drift (scenarios PD-TP-neap/spring) after entering the sea during neap on 3-January-2012 at 15:00 and spring tide on 7-January-2012 at 12:00

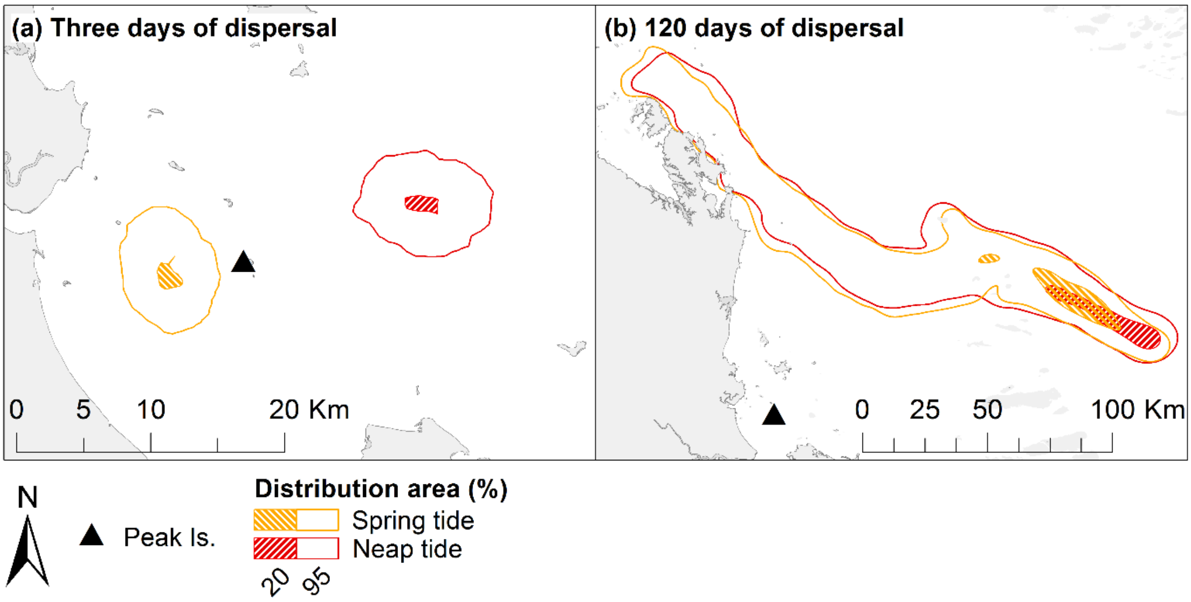

Supplement: Figure S2. S-flatback distribution probabilities from Peak Island after (a) 3 days and (b) 120 days of passive drift (scenarios PD-TP-neap/spring) [file rsos170164supp2.pdf]
