# Supplementary material for: Does behaviour affect the dispersal of flatback post-hatchlings in the Great Barrier Reef?
Source: R Soc Open Sci. 2017 May 24;4(5):170164. doi: 10.1098/rsos.170164 (PMC5451825; doi:10.1098/rsos.170164)

Royal Society Open Science

The following supplement accompanies the article

Does behaviour affect the dispersal of flatback post-hatchlings in the Great Barrier Reef?

Natalie Wildermann[1, 2], Kay Critchell[1, 2], Mariana MPB Fuentes[3], Colin Limpus[4], Eric Wolanski[1, 2], and Mark Hamann[1, 2].

http://dx.doi.org/10.1098/rsos.170164

Author for correspondence:
Natalie Wildermann
E-mail: natalie.wildermann@my.jcu.edu.au

Figure S3. Dispersal success (percentage of s-flatbacks in inshore waters) for each of the swimming parameters we evaluated in the sensitivity analysis: (a) swimming speed, (b) swimming direction, (c) proportion of time swimming per day, and (d) the day the post-frenzy swimming behaviour started. For each scenario, the dispersal success for Wild Duck Island, Peak Island. Dispersal success are Very low (≤ 25%), Low (25.01 – 50%), Medium (51.01 – 75%) and High (> 75%). For all scenarios s-flatbacks were modelled for 120 days until 00h on 2nd May 2012.

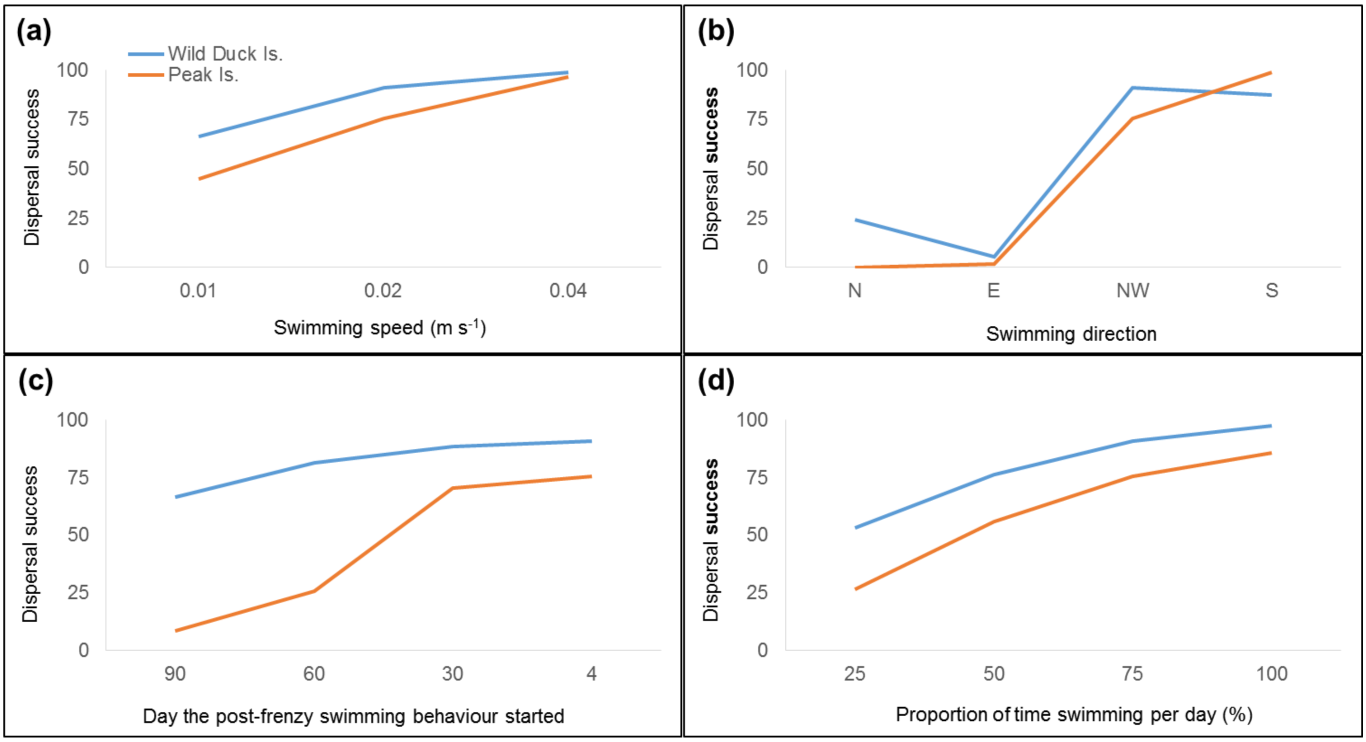

Supplement: Figure S3. Dispersal success (percentage of s-flatbacks in inshore waters) for each of the swimming parameters we evaluated in the sensitivity analysis: (a) swimming speed, (b) swimming direction, (c) proportion of time swimming per day, and (d) the day the post-frenzy swimming behaviour started. .  [file rsos170164supp3.pdf]
