# Supplementary material for: Does behaviour affect the dispersal of flatback post-hatchlings in the Great Barrier Reef?
Source: R Soc Open Sci. 2017 May 24;4(5):170164. doi: 10.1098/rsos.170164 (PMC5451825; doi:10.1098/rsos.170164)
Supplement: Table S1. Summary of releasing locations of s-turtles [file rsos170164supp4.pdf]

Royal Society Open Science

The following supplement accompanies the article

Does behaviour affect the dispersal of flatback post-hatchlings in the Great Barrier Reef?

Natalie Wildermann[1, 2], Kay Critchell[1, 2], Mariana MPB Fuentes[3], Colin Limpus[4], Eric Wolanski[1, 2], and Mark Hamann[1, 2].

http://dx.doi.org/10.1098/rsos.170164

Author for correspondence:
Natalie Wildermann
E-mail: natalie.wildermann@my.jcu.edu.au

Table S1. Summary of releasing locations of s-turtles. BS: Broad Sound, C: Capricornia, CBG: Capricorn Bunker Group, Nd: flatback (*Natator depressus*), Cm: green (*Chelonia mydas*), Cc: loggerhead (*Caretta caretta*), PD: passive drift, GL: geographic location, minor: minor rookery, major: major rookery, non: non-nesting beach.

**Region**	**Type of rookery**	**Locality**	**Coordinates**		**Nesting species**	**Scenarios**
BS	Major	Wild Duck Is.	149.859	-22.001	Nd	All (see Table 1)
BS	Minor/ Intermediate	Avoid Is.: SE East Beach<br>Long Is.: East Beach<br>Swb Beach North Side Mcdonald Pt.<br>Swb 2nd Beach South of Stanage Beach<br>Infelix Islets: South<br>Lingham: Northern Beach<br>Red Clay Isle: Western Beach	149.664<br>149.907<br>150.185<br>150.082<br>149.841<br>150.263<br>149.646	-21.977<br>-22.083<br>-22.319<br>-22.166<br>-22.033<br>-22.226<br>-21.930	Nd	PD-GL-species,<br>PD-GL-regions,<br>PD-GL-minor/major
BS	Non-nesting	Red Clay Isle: East Beach<br>Wild Duck Is.: South Beach<br>Swb Bat Cave Beach,South<br>Swb Sth of Yenyarindle Hut<br>Tin Case Ck Southward<br>West Side Island<br>Marble Island	149.651<br>149.864<br>150.061<br>150.142<br>149.541<br>149.849<br>150.151	-21.936<br>-22.005<br>-22.140<br>-22.255<br>-22.128<br>-22.149<br>-21.980	-	PD-GL-non/major
C	Major	Peak Is.	150.932	-23.341	Nd	All (see Table 1)
C	Minor/ Intermediate	Facing Is.: North Beach<br>Facing Is.: Settlement Bay<br>Stockyard Point<br>Emu Pt.: Tanby Pt.<br>Wild Cattle Island<br>Curtis Is.: Southend<br>NWGKIs Big Pen – 3rd beach	151.341<br>151.388<br>150.827<br>150.820<br>151.413<br>151.258<br>150.945	-23.783<br>-23.869<br>-22.691<br>-23.232<br>-23.973<br>-23.665<br>-23.171	Nd	PD-GL-species,<br>PD-GL-regions,<br>PD-GL-minor/major
C	Non-nesting	Hummock Hill Is.<br>Middle Is.<br>Curtis Is: Cape Keppell<br>Humpy Island<br>North Keppell<br>Water Park Point<br>Boyne Island	151.467<br>151.740<br>151.057<br>150.966<br>150.900<br>150.770<br>151.324	-23.996<br>-24.000<br>-23.449<br>-23.216<br>-23.070<br>-22.940<br>-23.873	-	PD-GL-non/major
CBG	Major/Minor	Wreck Is.<br>North West Is.<br>Heron Is.	151.968<br>151.710<br>151.885	-23.314<br>-23.274<br>-23.448	Cm, Cc	PD-GL-species
